# Harnessing PM2.5 Exposure Data to Predict Progression of Fibrotic Interstitial Lung Diseases Based on Telomere Length

**DOI:** 10.3389/fmed.2022.871898

**Published:** 2022-05-12

**Authors:** Jessica Germaine Shull, Lurdes Planas-Cerezales, Carla Lara Compte, Rosario Perona, Maria Molina-Molina

**Affiliations:** ^1^Interstitial Lung Disease (ILD) Multidisciplinary Unit, Hospital Universitari Bellvitge, Instituto de Investigación Biomédica de Bellvitge (IDIBELL), Universitat de Barcelona, Hospitalet de Llobregat, L'Hospitalet de Llobregat, Spain; ^2^Instituto de Investigaciones Biomedicas Consejo Superior de Investigaciones Científicas/Universidad Autónoma de Madrid (CSIC/UAM), Madrid, Spain; ^3^Centro Investigación Biomédica en Red de Enfermedades Raras, Instituto de Salud Carlos III, Madrid, Spain; ^4^Centro Investigación Biomédica en Red de Enfermedades Respiratorias, Instituto de Salud Carlos III, Madrid, Spain

**Keywords:** pulmonary fibrosis, pollution, telomeres, big data, impact PM2.5

## Abstract

Cross-analysis of clinical and pollution factors could help calculate the risk of fibrotic interstitial lung disease (ILD) development and progression. The intent of this study is to build a body of knowledge around early detection and diagnosis of lung disease, harnessing new data sets generated for other purposes. We cross-referenced exposure levels to particulate matter 2.5 (PM2.5) with telomere length of a cohort of 280 patients with fibrotic ILD to weigh impact and associations. There was no linear correlation between PM2.5 and telomere length in our data sets, as the value of the correlation coefficient was 0.08. This exploratory study offers additional insights into methodologies for investigating the development and prognosis of pulmonary fibrosis.

## Introduction

Several characteristics have been associated with an increased risk of fibrotic interstitial lung disease (ILD) development, such as smoking, viral infections, existence of familial aggregation, and telomere dysfunction (1–4). Across all fibrotic ILDs, patients with shortened telomeres have a more accelerated disease progression. Research has also shown that air pollution has a direct effect on lung disease (5). Particulate matter with an aerodynamic diameter of ≤ 2.5 μm (PM2.5) is the smallest particulate matter for which we have long-term exposure estimates in Catalonia, and because of its size, it is one of the pollutants that can most easily reach the deepest tissue and alveoli of the lungs. One systematic review of more than 12,000 subjects across 25 studies found associations between air pollution and telomere shortening (6), and PM2.5 has been suggested as a possible cause of COPD in studies as early as 2014 (7). In addition, it has been shown that exposure to PM2.5 resulted in shortened telomeres and altered telomerase activity in human bronchial epithelial cells (8).

Given this background, this study cross-analyzed telomere length and exposure to PM2.5 to determine associations between these known risk factors in our cohort of patients with fibrotic ILD.

## Methods

In this retrospective study, we analyzed a cohort of 280 patients with fibrotic ILD in the northeast region of Spain who were evaluated for telomere length (TL) in our center because of the indication of the potential risk of telomere shortening from 2014 to 2020. The Ethics Committee of Hospital Universitari de Bellvitge (HUB) approved the study, and all the patients provided written informed consent before inclusion. The relative telomere length was assessed at the time of diagnosis by quantitative polymerase chain reaction (qPCR), as previously described (9). Since telomere length changes with age, a Z-score value was obtained to allow for comparisons of telomere length among individuals of different ages and among cohorts (10). The Z-score compares the telomere shortening ratio value in each individual with the age-matched mean and standard deviation (SD) of the values obtained in the controls. A Z-score below the 10th percentile of a normal distribution was considered as severe telomere shortening. In the statistical analysis, a description of the baseline and clinical characteristics of the patients was made according to their distribution. A linear model was estimated using forced vital capacity (FVC) at 3 years as the dependent variable and using baseline forced vital capacity, exposure to PM2.5, industrial exposure, age, and sex as variables of interest.

Disease progression was considered when a patient presented at least two of the following criteria in the absence of any other explanation or cause: (a) worsening of respiratory symptoms, (b) physiological progression [absolute decline in the FVC of ≥10% or DLCO (corrected for Hb) of ≥15%], and (c) death. FVC value, over time, was used to analyze potential correlations.

The population analyzed for disease progression was generally older (average age 64.8) and of Spanish nationality. We used their current postal code as the location variable, because the tendency in this population to change residence is very low. Survey data from the province of Barcelona in 2006 show that the age at which people change residence is primarily between 25 and 40 years old; after the age of 60, the likelihood and desire to move is 2–6% (11). According to this survey, 75% of people over 60 in Catalonia believe that where they currently live is the best place to live and that number increases to 81% after the age of 75. Refer Supplementary Data 2 for further information.

**Figure 1:**
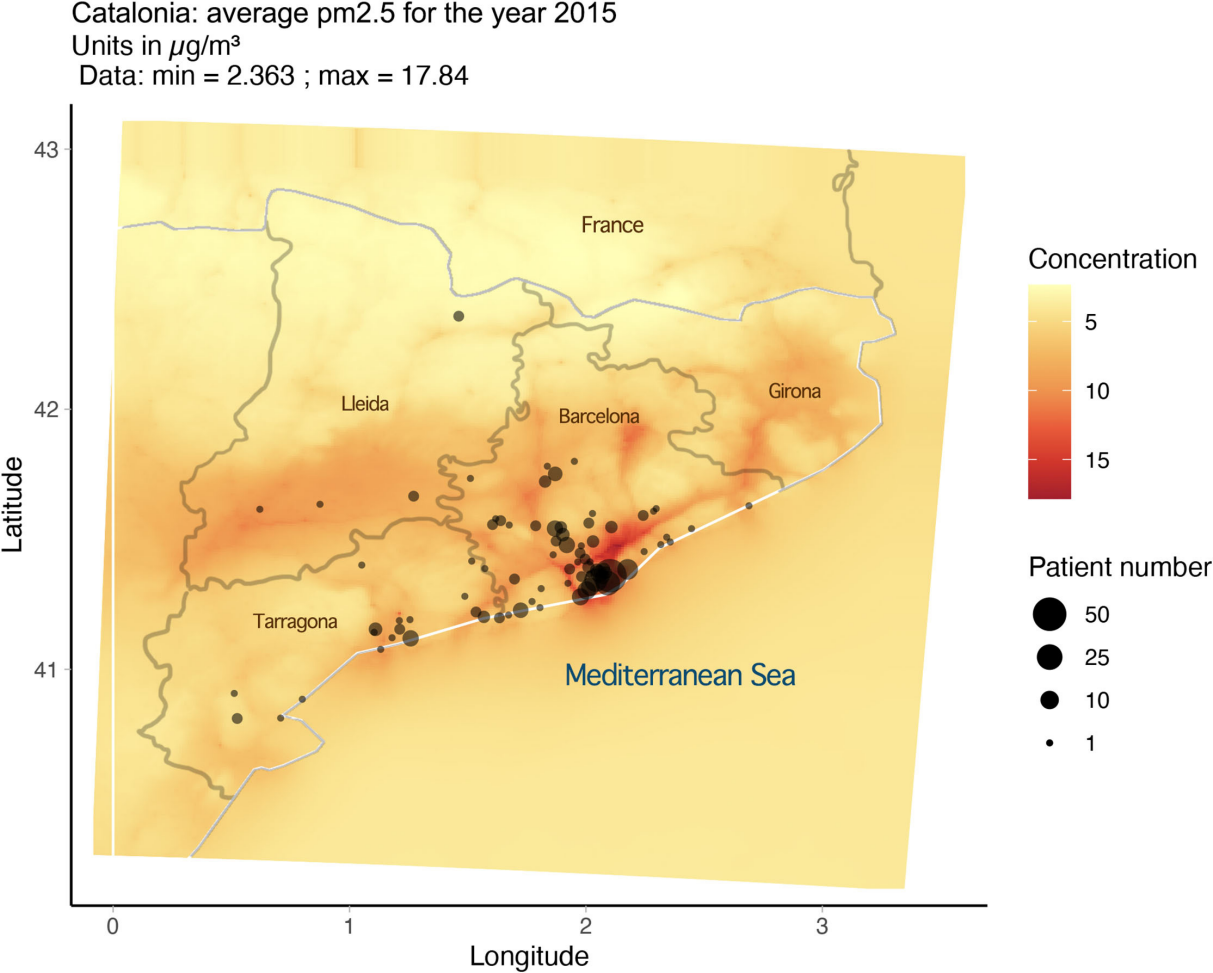
Map of Catalonia showing average PM2.5 exposure over one year with patient location.

PM2.5 exposure and variables were derived from data from the CALIOPE modeling system (12–15), which has been positively evaluated for epidemiological research (12). As noted in our previous publication (16), the average exposure for PM2.5 for any year in any 1-km area remained consistent from 2001 to 2017. Based on this, the estimates of PM2.5 exposure for 2015 were utilized and extrapolated to serve as an estimate for long-term exposure (2001–2017). Approximate locations for the 280 patients were based on postal code and then plotted using R (version 3.6.0) and the Google Map API and superimposed over the exposure map (Figure 1). From this mapping exercise, we can assign an approximate level of long-term exposure to PM2.5 for each subject in the cohort. With the data gathered, we ran a statistical analysis on PM2.5 exposure and Z-score for the cohort.

A complete table of the average PM2.5 exposure and Z-score for each patient can be seen in Supplementary Data 1.

## Results

The diagnosis for the 280 fibrotic ILD cases was primarily IPF, with 138 cases or 49.2% of the total participants; the next largest number of cases was fibrotic forms of hypersensitivity pneumonitis (HP) with 32 cases or 11.4%. Unclassifiable ILDs (uILD) and the related interstitial pneumonia with autoimmune features (IPAF) formed the third largest group at 26 cases or 9.2% of the participants, followed by diagnoses such as CTD-ILD (24 or 8.5%), non-specific interstitial pneumonia (NSIP) (18 or 6.4 %), smoking-related interstitial fibrosis (SRIF) (11 or 3.9%), fibrosis with organizing pneumonia (6 or 2.1%), and sarcoidosis IV (4 or 1.4%). The remaining cases were other fibrotic ILDs. Twenty-nine of the 280 referred family aggregation and 84 presented severe telomere shortening (20 of them had some pathogenic telomere-related gene mutation in RTEL1, TERT, TERC, or DKC1).

**Figure 2:**
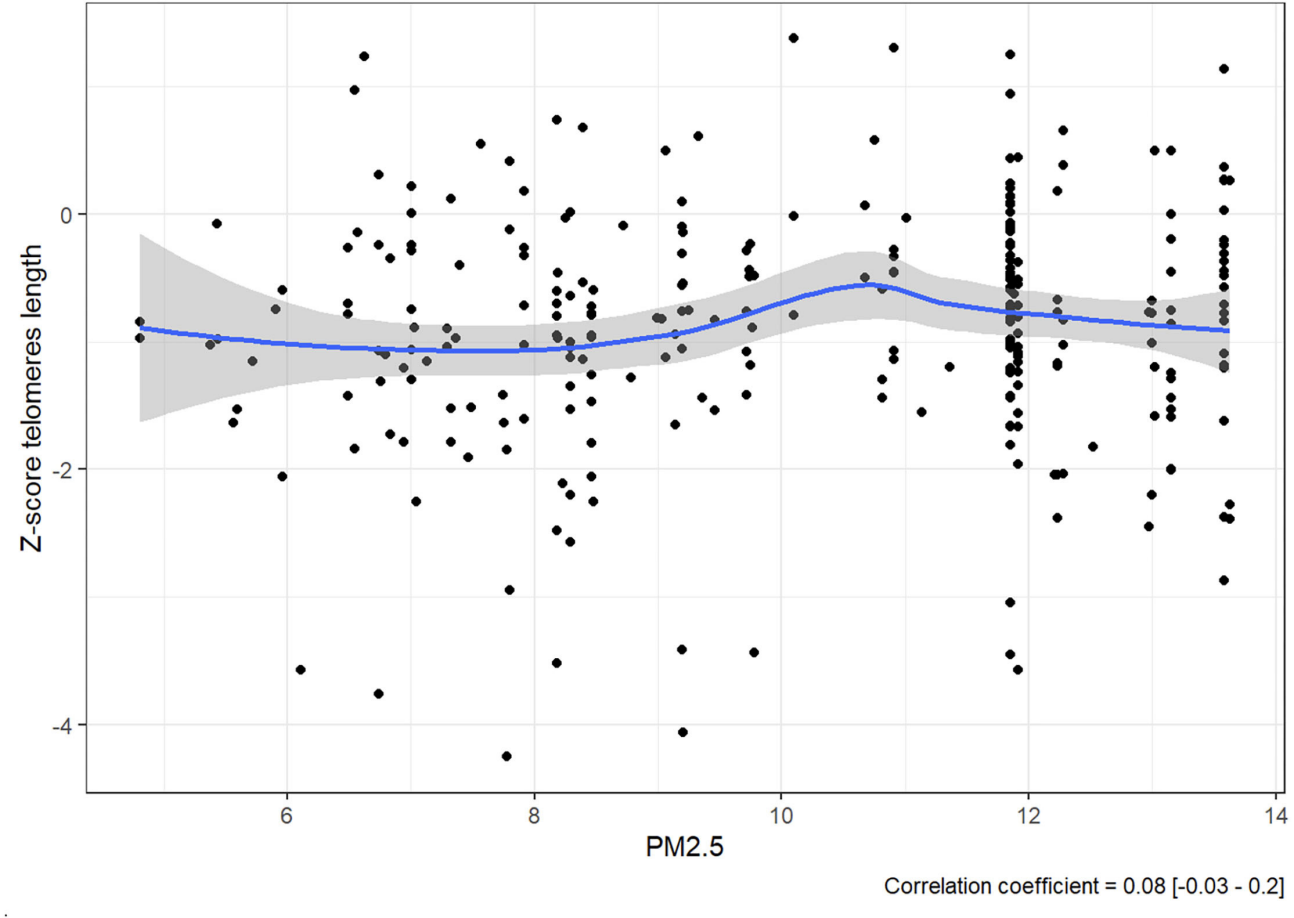
Average exposure PM2.5 plotted against Z-score in our cohort of 280 patients.

The expectation was to see evidence that consistent exposure to higher levels of PM2.5 was correlated to lower Z-score. However, rather than a steady decline in Z-score as PM2.5 increases, there is no linear correlation between them since the value of the correlation coefficient was 0.08 [−0.03, 0.2] (Figure 2).

There is, however, an accumulation of cases at the 12 μg/m3 line, and it should be noted that all the 280 members of the cohort were exposed consistently to PM2.5 at levels between 5.565 and 13.631 μg/m3.

## Discussion

In 2021, the WHO published updated Air Quality Guidelines (AQG) for PM2.5 as well as other hazardous airborne pollutants. The guidelines, based on data for cause-specific mortality, lead to a recommendation of long-term exposure to PM2.5 at levels of no more than 5 μg/m3 (17). This update means that every subject in the cohort was exposed to levels of PM2.5 above the WHO recommended level.

This observational study is not intended to be conclusive, and further research should be conducted with more specific individual measurements of exposure to PM2.5 and other airborne pollutants; however, we utilized the best data available.

In a more elucidative step, we then analyzed the progression of disease in the 84 patients with severe telomere shortening. Eighty cases had the necessary data available (4 did not complete the 2nd FVC measurement) and were documented with a Z-score in the 10th and 1st percentiles. We then compared their forced vital capacity (FVC) results at baseline and after 3 years. These factors were modeled using a multivariate linear model (Table 1). The result of the estimated model of FVC after 3 years using PM2.5 as a predictor was non-significant; however, Z-score was indicative of progression. For every one unit decrease in Z-score, the FVC measure at 3 years after baseline decreased by approximately 7 points. Twenty-eight of the 80 analyzed were smokers, although this did not correlate to telomere shortening. We also compared the diagnosis of the 80 cases, and the numbers were similar to the larger group: of the 80 cases, 40 (50%) were IPF. In this retrospective study, it was not possible to determine at what point in each subject's life environmental pollutants might have begun to affect lung tissue.

**Table 1:** The covariable “Z-score” is significant in the “Multivariate 1” model.

	**Univariate**				**Multivariate 1**		
**Predictors**	**Estimates**	**std. Error**	**CI**	***p***	**Estimates**	**std. Error**	**CI**	***P***
(Intercept)	87.11	11.13	64.83–109.38	<0.001	8.09	9.26	−10.45–26.63	0.386
Z-score	2.54	5.74	−8.95–14.02	0.660	6.92	3.21	0.49–13.34	0.035
Baseline FVC					1.02	0.09	0.84–1.20	<0.001
Observations	60			60		
*R*^2^/*R*^2^ adjusted	0.003/−0.014			0.698/0.688		
AIC	572.819			503.132		

*For every one unit decrease in the Z-score, the forced vital capacity at 3 years (FVC-3y) decreases by about 7 points*.

A thorough retrospective analysis with multiple risk factors weighted for impact could provide further insight into disease progression in patients with fibrosing ILDs. The long-term objective is to gain further insights into disease development and early diagnosis of ILDs by harnessing big data and analyzing risk factors with additional innovative methodologies.

## Data Availability Statement

The original contributions presented in the study are included in the article/Supplementary Material, further inquiries can be directed to the corresponding author/s.

## Ethics Statement

The Ethics Committee of Hospital Universitari de Bellvitge (HUB) approved the study and all patients provided written informed consent before inclusion.

## Author Contributions

JS was the senior author. MM-M was the authority in review and has last authorship. LP-C and RP contributed equally. CL contributed the essential mapping imagery. All the authors contributed valuable contents to this manuscript.

## Funding

This study was funded by the Instituto de Salud Carlos III through project PI18/00346 (co-funded by European Regional Development Fund, ERDF, a way to build Europe), AC19/00006 (projects of International Programs, ISCIII, Spain, supported by FEDER funds), the Spanish Society of Respiratory (SEPAR), the Barcelona Respiratory Network (BRN), the Fundació Ramón Pla Armengol, ISCIII PI18/00346, and Boehringer Ingelheim.

## Conflict of Interest

MM-M has received grants and fees for research advice from Roche, Esteve-Teijin, and Boehringer Ingelheim. The remaining authors declare that the research was conducted in the absence of any commercial or financial relationships that could be construed as a potential conflict of interest. The reviewer PR declared a past co-authorship with the authors JS and MM-M to the handling editor.

## Publisher's Note

All claims expressed in this article are solely those of the authors and do not necessarily represent those of their affiliated organizations, or those of the publisher, the editors and the reviewers. Any product that may be evaluated in this article, or claim that may be made by its manufacturer, is not guaranteed or endorsed by the publisher.

## Supplementary Material

Supplementary Table 1 Cohort of 280 subjects with age, gender, telomere length, and individual exposure level to PM2.5 in microns.

## Abbreviations

AQG: Air Quality Guidelines
COPD: chronic obstructive pulmonary disease
CTD-ILD: connective tissue disease- interstitial lung disease
FVC: forced vital capacity
HP: hypersensitivity pneumonitis
IPAF: interstitial pneumonia with autoimmune features
IPF: idiopathic pulmonary fibrosis
ILD: interstitial lung disease
NSIP: nonspecific interstitial pneumonia
PM 2.5: particulate matter with an aerodynamic diameter of ≤ 2.5 μm
qPCR: quantitative polymerase chain reaction
uILD: unclassifiable interstitial lung disease
WHO: World Health Organization.
