# Supplementary material for: Harnessing PM2.5 Exposure Data to Predict Progression of Fibrotic Interstitial Lung Diseases Based on Telomere Length
Source: Front Med (Lausanne). 2022 May 12;9:871898. doi: 10.3389/fmed.2022.871898 (PMC9133476; doi:10.3389/fmed.2022.871898)
Supplement: Supplementary Table 1 — Cohort of 280 subjects with age, gender, telomere length, and individual exposure level to PM2.5 in microns. [file Table_1.PDF]

Gender	Age at swab	Telomere length	Severe telomere shortening	Post code	Occupational Exposure	Latitude	Longitude	Province in Catalonia	Exposition to PM2p5 in µg/m3	PM2p5 color key - reference for Fig 1
F	53	1,022,031,219	yes	08001		413,842,466	217,634,927	Barcelona	13,151,708	
M	69	947,413,106	yes	08011		413,842,466	217,634,927	Barcelona	13,151,708	
F	62	873,522,067	yes	08014		413,842,466	217,634,927	Barcelona	13,151,708	
M	59	894,065,323	yes	08016		413,842,466	217,634,927	Barcelona	13,151,708	
F	53	895,395,168	yes	08016		413,842,466	217,634,927	Barcelona	13,151,708	
M	68	1,344,251,452	no	08017		413,842,466	217,634,927	Barcelona	13,151,708	
F	58	1,575,910,329	no	08017		413,842,466	217,634,927	Barcelona	13,151,708	
M	77	1,338,266,127	no	08023		413,842,466	217,634,927	Barcelona	13,151,708	
M	63	817,013,181	yes	08025		413,842,466	217,634,927	Barcelona	13,151,708	
F	66	1,417,926,211	no	08028		413,842,466	217,634,927	Barcelona	13,151,708	
F	48	1,192,049,668	yes	08028		413,842,466	217,634,927	Barcelona	13,151,708	
M	59	1,233,499,467	no	08029		413,842,466	217,634,927	Barcelona	13,151,708	
F	66	109,483,749	no	08029		413,842,466	217,634,927	Barcelona	13,151,708	
M	71	957,059,422	yes	08042		413,842,466	217,634,927	Barcelona	13,151,708	
M	76	1,466,001,641	no	08185		415,915,542	224,224,029	Barcelona	10,678,108	
F	51	130,306,665	no	08185		415,915,542	224,224,029	Barcelona	10,678,108	
F	85	1,273,879,494	no	08191		41,491,737	203,003,252	Barcelona	10,098,553	
F	49	1,346,061,857	no	08191		41,491,737	203,003,252	Barcelona	10,098,553	
M	64	1,939,686,716	no	08191		41,491,737	203,003,252	Barcelona	10,098,553	
M	72	890,842,477	yes	08206		41,546,959	210,778,994	Barcelona	13,631,563	
M	71	862,145,215	yes	08206		41,546,959	210,778,994	Barcelona	13,631,563	
M	72	1,514,982,673	no	08206		41,546,959	210,778,994	Barcelona	13,631,563	
M	80	1,465,565,139	no	08220		413,425,371	204,182,116	Barcelona	9,331,097	
M	69	1,606,464,596	no	08222		415,623,635	201,215,141	Barcelona	9,060,313	
F	78	1,172,919,739	no	08222	1	415,623,635	201,215,141	Barcelona	9,060,313	
F	64	1,132,534,656	no	08230		415,992,176	2,027,681	Barcelona	9,247,734	
M	77	1,075,882,893	yes	08242	1	417,219,959	182,721,026	Barcelona	7,325,977	
F	42	103,831,059	yes	08242		417,219,959	182,721,026	Barcelona	7,325,977	
M	61	1,463,868,323	no	08242		417,219,959	182,721,026	Barcelona	7,325,977	
M	42	1,109,976,962	yes	08251	1	417,808,385	183,671	Barcelona	11,134,117	
F	68	1,407,795,816	no	08271		41,799,633	195,102,866	Barcelona	8,251,802	
M	44	1,224,488,392	yes	08272	1	417,515,078	187,003,405	Barcelona	9,752,857	
M	58	137,490,904	no	08272	1	417,515,078	187,003,405	Barcelona	9,752,857	
M	77	1,335,199,397	no	08272		417,515,078	187,003,405	Barcelona	9,752,857	
F	64	801,070,344	yes	08280		417,340,027	15,114,597	Barcelona	7,757,558	
M	71	611,468,079	yes	08292	1	415,412,375	186,885,891	Barcelona	9,199,032	
M	64	1,455,681,305	no	08292		415,412,375	186,885,891	Barcelona	9,199,032	
M	72	1,425,642,928	no	08292	1	415,412,375	186,885,891	Barcelona	9,199,032	
M	57	1,151,801,428	no	08292		415,412,375	186,885,891	Barcelona	9,199,032	
F	68	1,208,163,062	no	08292		415,412,375	186,885,891	Barcelona	9,199,032	
M	75	1,373,045,621	no	08292		415,412,375	186,885,891	Barcelona	9,199,032	
M	51	1,230,965,613	no	08292		415,412,375	186,885,891	Barcelona	9,199,032	
M	66	1,233	no	08293		417,515,078	187,003,405	Barcelona	973,979	
F	68	1,252,740,628	no	08293		417,515,078	187,003,405	Barcelona	973,979	
F	52	91,933,096	yes	08303		415,408,469	244,658,062	Barcelona	7,465,206	
M	65	845,539,469	yes	08320		414,794,376	231,648,978	Barcelona	7,489,675	
M	70	896,142,153	yes	08330	1	414,896,047	235,662,125	Barcelona	7,048,431	
F	56	1,330,789,323	no	08338		415,080,205	234,158,045	Barcelona	7,399,726	
M	64	1,364,168,783	no	08397		416,287,647	268,966,552	Barcelona	6,572,365	
M	74	1,296,275,194	no	08402		416,079,309	2,286,177	Barcelona	11,887,263	
M	68	645,078,783	yes	08520		416,177,892	22,971,592	Barcelona	1,220,823	
F	66	82,111,829	yes	08620	1	413,927,246	201,065,601	Barcelona	13,019,416	
M	71	115,550,465	no	08620	1	413,927,246	201,065,601	Barcelona	13,019,416	
F	70	1,572,254,409	no	08620		413,927,246	201,065,601	Barcelona	13,019,416	
M	57	414,018,444	yes	08629		413,567,096	19,813,139	Barcelona	6,742,324	
M	70	1,185,962,408	no	08629		413,567,096	19,813,139	Barcelona	6,742,324	
F	65	1,132,221,066	no	08630		415,179,435	190,221,018	Barcelona	9,715,393	
F	47	1,152,857,433	yes	08630	1	415,179,435	190,221,018	Barcelona	9,715,393	
M	43	1,257,633,439	no	08630		415,179,435	190,221,018	Barcelona	9,715,393	
M	75	1,378,837,274	no	08630		415,179,435	190,221,018	Barcelona	9,715,393	
F	59	170,379,401	no	08635		414,933,138	187,385,015	Barcelona	6,544,985	
M	73	996,958,212	yes	08635		414,933,138	187,385,015	Barcelona	6,544,985	
M	37	454,815,909	yes	08640	1	415,458,402	189,387,309	Barcelona	9,204,964	
M	67	1,364,422,672	no	08640		415,458,402	189,387,309	Barcelona	9,204,964	
F	76	131,632,908	no	08640		415,458,402	189,387,309	Barcelona	9,204,964	
M	62	728,654,674	yes	08690		413,663,146	201,483,385	Barcelona	12,523,562	
M	52	1,413,686,143	no	08700		41,578,951	161,829,588	Barcelona	8,722,155	
M	56	1,155,245,008	no	08710	1	41,556,596	160,489,809	Barcelona	7,293,052	
M	65	1,081,089,568	no	08710		41,556,596	160,489,809	Barcelona	7,293,052	
M	83	596,313,589	yes	08720	1	413,472,121	16,977,467	Barcelona	8,475,286	
M	84	109,933,281	no	08720	1	413,472,121	16,977,467	Barcelona	8,475,286	
F	39	1,108,149,338	yes	08737	1	413,873,941	157,242,423	Barcelona	5,565,649	

M	73	1,197,081,912	no	08738		414,161,012	151,689,813	Barcelona	5,379,079	
M	68	1,104,529,825	no	08740	1	414,467,566	197,523,925	Barcelona	9,463,125	
F	75	1,071,418,892	yes	08740		414,467,566	197,523,925	Barcelona	9,463,125	
M	63	966,625,922	no	08750		414,132,111	201,736,908	Barcelona	11,365,413	
M	58	1,089,221,362	yes	08755		414,748,609	198,046,176	Barcelona	8,780,173	
M	59	1,776,788,124	no	08756		414,128,118	196,561,405	Barcelona	662,063	
M	63	1,326,801,476	no	08759	1	413,854,293	193,107,649	Barcelona	6,737,562	
F	52	1,523,105,346	no	08759		413,854,293	193,107,649	Barcelona	6,737,562	
M	49	1,991,007,605	no	08760		414,780,033	191,913,747	Barcelona	10,912,683	
F	64	1,248,554,514	no	08760		414,780,033	191,913,747	Barcelona	10,912,683	
M	37	1,424,846,425	no	08760		414,780,033	191,913,747	Barcelona	10,912,683	
M	70	138,132,614	no	08760	1	414,780,033	191,913,747	Barcelona	10,912,683	
M	74	1,169	no	08760		414,780,033	191,913,747	Barcelona	10,912,683	
M	65	1,014,540,732	no	08770		414,780,033	191,913,747	Barcelona	10,912,683	
M	62	1,293,846,862	no	08770		414,780,033	191,913,747	Barcelona	10,912,683	
F	72	1,260,364,419	no	08780	1	414,241,248	199,610,661	Barcelona	1,297,574	
M	48	833,325,404	yes	08780		414,241,248	199,610,661	Barcelona	1,297,574	
M	52	111,070,918	yes	08781	1	415,519,192	178,743,519	Barcelona	6,944,502	
M	52	952,662,046	yes	08781		415,519,192	178,743,519	Barcelona	6,944,502	
M	60	1,197,715,769	yes	08787		415,548,414	16,755,747	Barcelona	9,765,711	
M	33	622,930,813	yes	08788		41,571,618	163,933,412	Barcelona	9,779,184	
M	63	1,237,100,764	no	08788	1	41,571,618	163,933,412	Barcelona	9,779,184	
F	52	1,139,886,351	no	08790		414,405,341	186,136,831	Barcelona	6,794,488	
M	67	1,295,720,773	no	08800	1	412,272,199	17,238,399	Barcelona	792,188	
M	54	1,367,009,658	no	08800		412,272,199	17,238,399	Barcelona	792,188	
M	69	1,147,270,017	no	08800		412,272,199	17,238,399	Barcelona	792,188	
F	72	1,055,803,306	yes	08800		412,272,199	17,238,399	Barcelona	792,188	
M	52	1,159,345,485	no	08800		412,272,199	17,238,399	Barcelona	792,188	
F	66	1,486,337,586	no	08800		412,272,199	17,238,399	Barcelona	792,188	
M	48	1,232,754,523	no	08812		412,606,932	177,197,485	Barcelona	7,128,533	
M	66	983,752,847	no	08818	1	413,103,609	181,063,174	Barcelona	5,721,355	
M	87	1,624,205,068	no	08820		413,309,585	20,926,236	Barcelona	13,582,034	
M	66	1,560,070,264	no	08820		413,309,585	20,926,236	Barcelona	13,582,034	
M	75	866,036,925	yes	08820		413,309,585	20,926,236	Barcelona	13,582,034	
M	65	963,901,341	no	08820		413,309,585	20,926,236	Barcelona	13,582,034	
M	75	1,258,443,155	no	08820	1	413,309,585	20,926,236	Barcelona	13,582,034	
M	65	1,151,369,643	no	08820	1	413,309,585	20,926,236	Barcelona	13,582,034	
M	67	152,282,068	no	08820		413,309,585	20,926,236	Barcelona	13,582,034	
F	76	744,508,753	yes	08820	1	413,309,585	20,926,236	Barcelona	13,582,034	
M	78	1,514,539,296	no	08820	1	413,309,585	20,926,236	Barcelona	13,582,034	
F	54	1,337,691,692	no	08820		413,309,585	20,926,236	Barcelona	13,582,034	
M	57	1,141,035,606	no	08820		413,309,585	20,926,236	Barcelona	13,582,034	
F	55	997,161,056	yes	08820		413,309,585	20,926,236	Barcelona	13,582,034	
M	82	1,104,727,588	no	08820	1	413,309,585	20,926,236	Barcelona	13,582,034	
F	67	1,236,510,879	no	08820		413,309,585	20,926,236	Barcelona	13,582,034	
M	66	1,103,633,766	no	08820		413,309,585	20,926,236	Barcelona	13,582,034	
M	68	130,107,421	no	08820	1	413,309,585	20,926,236	Barcelona	13,582,034	
M	49	1,224,556,874	yes	08820		413,309,585	20,926,236	Barcelona	13,582,034	
M	66	1,341,829,991	no	08820	1	413,309,585	20,926,236	Barcelona	13,582,034	
M	62	1,251,974,163	no	08820	1	413,309,585	20,926,236	Barcelona	13,582,034	
M	68	1,430,908,383	no	08820		413,309,585	20,926,236	Barcelona	13,582,034	
F	61	1,327,985,738	no	08820		413,309,585	20,926,236	Barcelona	13,582,034	
M	73	968,710,434	yes	08820		413,425,371	204,182,116	Barcelona	11,915,233	
F	67	82,922,458	yes	08830		413,425,371	204,182,116	Barcelona	11,915,233	
F	67	1,008,912,304	no	08830		413,425,371	204,182,116	Barcelona	11,915,233	
M	55	1,184,871,373	no	08830		413,425,371	204,182,116	Barcelona	11,915,233	
M	69	1,210,883,054	no	08830		413,425,371	204,182,116	Barcelona	11,915,233	
M	61	1,224,550,764	no	08830	1	413,425,371	204,182,116	Barcelona	11,915,233	
M	68	78,989,962	yes	08830	1	413,425,371	204,182,116	Barcelona	11,915,233	
F	50	465,484,119	yes	08830		413,425,371	204,182,116	Barcelona	11,915,233	
M	61	111,469,687	no	08830	1	413,425,371	204,182,116	Barcelona	11,915,233	
M	60	996,192,467	no	08830		413,425,371	204,182,116	Barcelona	11,915,233	
M	54	1,123,825,503	yes	08830		413,425,371	204,182,116	Barcelona	11,915,233	
F	68	1,277,255,313	no	08830		413,425,371	204,182,116	Barcelona	11,915,233	
M	57	1,073,132,453	yes	08830		413,425,371	204,182,116	Barcelona	11,915,233	
M	75	1,558,352,807	no	08830	1	413,425,371	204,182,116	Barcelona	11,915,233	
M	70	119,400,287	no	08830		413,425,371	204,182,116	Barcelona	11,915,233	
F	73	114,613,109	no	08830		413,425,371	204,182,116	Barcelona	11,915,233	
M	72	1,273	no	08830		413,425,371	204,182,116	Barcelona	11,915,233	
M	75	1,118,794,423	yes	08840	1	413,173,253	201,824,715	Barcelona	8,290,411	
M	75	908,800,284	yes	08840		413,173,253	201,824,715	Barcelona	8,290,411	
M	39	855,561,198	yes	08840		413,173,253	201,824,715	Barcelona	8,290,411	
F	61	1,178,039,833	no	08840	1	413,173,253	201,824,715	Barcelona	8,290,411	
M	66	993,657,394	no	08840	1	413,173,253	201,824,715	Barcelona	8,290,411	
F	58	1,023,317,654	yes	08840		413,173,253	201,824,715	Barcelona	8,290,411	

M	59	1,166,451,688	no	08840		413,173,253	201,824,715	Barcelona	8,290,411	
M	66	1,023,030,683	no	08840	1	413,173,253	201,824,715	Barcelona	8,290,411	
F	65	1,424,311,879	no	08840		413,173,253	201,824,715	Barcelona	8,290,411	
M	49	1,295,731,072	no	08850		413,033,167	200,329,904	Barcelona	8,463,221	
M	66	1,119,807,672	no	08850		413,033,167	200,329,904	Barcelona	8,463,221	
M	63	862,102,073	yes	08850		413,033,167	200,329,904	Barcelona	8,463,221	
M	61	740,288,456	yes	08850		413,033,167	200,329,904	Barcelona	8,463,221	
M	74	943,248,676	yes	08850	1	413,033,167	200,329,904	Barcelona	8,463,221	
M	66	1,053,209,943	no	08850		413,033,167	200,329,904	Barcelona	8,463,221	
M	68	941,526,084	yes	08850		413,033,167	200,329,904	Barcelona	8,463,221	
F	66	1,145,168,214	no	08850		413,033,167	200,329,904	Barcelona	8,463,221	
M	73	1,259	no	08850		413,033,167	200,329,904	Barcelona	8,463,221	
M	69	1,136,721,148	no	08859		413,307,064	192,397,654	Barcelona	5,910,691	
M	74	1,266,844,884	no	08860		412,787,734	197,680,641	Barcelona	7,009,918	
M	67	929,402,457	yes	08860		412,787,734	197,680,641	Barcelona	7,009,918	
M	58	1,442,252,401	no	08860		412,787,734	197,680,641	Barcelona	7,009,918	
F	60	132,758,067	no	08860		412,787,734	197,680,641	Barcelona	7,009,918	
F	67	1,309,501,236	no	08860		412,787,734	197,680,641	Barcelona	7,009,918	
F	73	1,502,169,404	no	08860		412,787,734	197,680,641	Barcelona	7,009,918	
M	70	1,131,737,056	yes	08860		412,787,734	197,680,641	Barcelona	7,009,918	
M	79	1,188,423,616	no	08860		412,787,734	197,680,641	Barcelona	7,009,918	
M	59	1,082,712,074	yes	08870		412,371,694	180,568,423	Barcelona	675,436	
F	77	1,209,757,158	no	08880		412,087,258	167,282,672	Barcelona	7,364,448	
M	66	145,326,209	no	08901		413,597,705	210,138,287	Barcelona	11,854,266	
M	71	1,415,524,734	no	08901	1	413,597,705	210,138,287	Barcelona	11,854,266	
F	74	1,484,307,301	no	08901		413,597,705	210,138,287	Barcelona	11,854,266	
M	75	1,040,691,453	yes	08901		413,597,705	210,138,287	Barcelona	11,854,266	
F	68	1,495,434,807	no	08901		413,597,705	210,138,287	Barcelona	11,854,266	
M	73	1,556,950,312	no	08901		413,597,705	210,138,287	Barcelona	11,854,266	
M	71	1,143,098,352	no	08901	1	413,597,705	210,138,287	Barcelona	11,854,266	
F	78	1,005,308,682	yes	08901	1	413,597,705	210,138,287	Barcelona	11,854,266	
M	69	1,280,763,159	no	08901		413,597,705	210,138,287	Barcelona	11,854,266	
M	70	1,048,510,509	no	08901	1	413,597,705	210,138,287	Barcelona	11,854,266	
F	85	114,009,399	no	08901	1	413,597,705	210,138,287	Barcelona	11,854,266	
M	82	1,122,722,699	no	08901		413,597,705	210,138,287	Barcelona	11,854,266	
M	59	1,308,800,549	no	08901		413,597,705	210,138,287	Barcelona	11,854,266	
M	66	1,447,125,726	no	08901		413,597,705	210,138,287	Barcelona	11,854,266	
M	72	1,425,963,384	no	08901		413,597,705	210,138,287	Barcelona	11,854,266	
M	66	1,392	no	08901		413,597,705	210,138,287	Barcelona	11,854,266	
F	62	1,193,100,624	no	08902		413,597,705	210,138,287	Barcelona	11,854,266	
M	72	1,418,836,098	no	08902	1	413,597,705	210,138,287	Barcelona	11,854,266	
M	65	880,849,257	yes	08902	1	413,597,705	210,138,287	Barcelona	11,854,266	
M	59	1,237,285,963	no	08902		413,597,705	210,138,287	Barcelona	11,854,266	
F	73	1,680,032,594	no	08902		413,597,705	210,138,287	Barcelona	11,854,266	
M	65	872,979,136	yes	08902		413,597,705	210,138,287	Barcelona	11,854,266	
F	78	600,830,899	yes	08902	1	413,597,705	210,138,287	Barcelona	11,854,266	
M	50	1,305,559,091	no	08902		413,597,705	210,138,287	Barcelona	11,854,266	
F	65	113,135,025	no	08902		413,597,705	210,138,287	Barcelona	11,854,266	
M	77	1,205,517,585	no	08902		413,597,705	210,138,287	Barcelona	11,854,266	
F	78	1,192,897,932	no	08902		413,597,705	210,138,287	Barcelona	11,854,266	
M	78	1,041,217,106	yes	08903		413,597,705	210,138,287	Barcelona	11,854,266	
F	63	1,098,811,814	no	08904		413,597,705	210,138,287	Barcelona	11,854,266	
F	76	1,469,817,709	no	08904		413,597,705	210,138,287	Barcelona	11,854,266	
F	61	146,953,216	no	08905		413,597,705	210,138,287	Barcelona	11,854,266	
M	77	1,346,011,627	no	08905		413,597,705	210,138,287	Barcelona	11,854,266	
M	68	1,128,468,697	no	08905	1	413,597,705	210,138,287	Barcelona	11,854,266	
M	67	1,203,346,037	no	08905		413,597,705	210,138,287	Barcelona	11,854,266	
M	76	1,150,496,195	no	08905	1	413,597,705	210,138,287	Barcelona	11,854,266	
M	69	1,323,960,086	no	08905		413,597,705	210,138,287	Barcelona	11,854,266	
M	68	1,334,308,992	no	08905		413,597,705	210,138,287	Barcelona	11,854,266	
F	77	701,988,945	yes	08906		413,597,705	210,138,287	Barcelona	11,854,266	
M	65	1,031,732,492	no	08906		413,597,705	210,138,287	Barcelona	11,854,266	
M	58	1,218,289,959	no	08906		413,597,705	210,138,287	Barcelona	11,854,266	
F	73	1,247,286,855	no	08906		413,597,705	210,138,287	Barcelona	11,854,266	
M	68	1,424,452,605	no	08906		413,597,705	210,138,287	Barcelona	11,854,266	
M	62	1,295,832,182	no	08906		413,597,705	210,138,287	Barcelona	11,854,266	
F	62	961,687,414	yes	08906		413,597,705	210,138,287	Barcelona	11,854,266	
M	77	1,275,050,916	no	08906		413,597,705	210,138,287	Barcelona	11,854,266	
M	70	1,209,331,027	no	08907	1	413,597,705	210,138,287	Barcelona	11,854,266	
M	67	1,892,874,889	no	08907		413,597,705	210,138,287	Barcelona	11,854,266	
M	82	1,255,086,551	no	08907		413,597,705	210,138,287	Barcelona	11,854,266	
M	67	1,511,644,994	no	08908		413,597,705	210,138,287	Barcelona	11,854,266	
M	61	1,408,856,305	no	08911		414,526,248	224,625,368	Barcelona	1,101,035	
F	76	1,543,393,819	no	08940		413,554,693	207,013,202	Barcelona	12,280,267	

M	71	950,210,735	yes	08940	1	413,554,693	207,013,202	Barcelona	12,280,267	
M	69	1,031,923,955	no	08940		413,554,693	207,013,202	Barcelona	12,280,267	
F	41	1,332,864,767	no	08940		413,554,693	207,013,202	Barcelona	12,280,267	
F	73	1,610,927,632	no	08940		413,554,693	207,013,202	Barcelona	12,280,267	
F	78	1,304,805,176	no	08950		41,372,119	20,840,895	Barcelona	10,819,815	
M	75	1,131,423,961	yes	08950		41,372,119	20,840,895	Barcelona	10,819,815	
M	77	1,095,359,986	yes	08950	1	41,372,119	20,840,895	Barcelona	10,819,815	
M	67	987,141,069	no	08960		413,860,259	207,609,668	Barcelona	8,396,422	
M	61	1,676,426,479	no	08960		413,860,259	207,609,668	Barcelona	8,396,422	
M	68	1,216,601,665	no	08960	1	413,860,259	207,609,668	Barcelona	8,396,422	
M	80	1,076,137,229	no	08970		41,367,564	205,542,054	Barcelona	122,342	
M	79	1,493,240,904	no	08970		41,367,564	205,542,054	Barcelona	122,342	
M	83	923,643,897	yes	08970		41,367,564	205,542,054	Barcelona	122,342	
M	34	999,273,951	yes	08970		41,367,564	205,542,054	Barcelona	122,342	
M	70	1,156,210,254	no	08970		41,367,564	205,542,054	Barcelona	122,342	
F	69	1,127,353,238	no	08970		41,367,564	205,542,054	Barcelona	122,342	
F	53	789,740,816	yes	08970		41,367,564	205,542,054	Barcelona	122,342	
M	59	838,800,259	yes	08980	1	413,807,438	204,579,427	Barcelona	12,996,239	
F	64	103,790,541	no	08980		413,807,438	204,579,427	Barcelona	12,996,239	
M	54	1,254,823,327	no	08980	1	413,807,438	204,579,427	Barcelona	12,996,239	
M	62	112,466,971	no	08980		413,807,438	204,579,427	Barcelona	12,996,239	
M	50	1,598	no	25001		416,152,736	62,061,934	Lleida	10,750,096	
M	73	404,460,839	yes	25200	1	416,657,554	127,119,164	Lleida	777,501	
F	54	935,317,399	yes	25200		416,657,554	127,119,164	Lleida	777,501	
M	72	1,094,917,199	yes	25244		416,345,458	87,512,433	Lleida	9,360,488	
F	47	1,328,173,166	no	25700		423,577,089	146,197,947	Lleida	4,809,573	
M	48	1,289,659,126	no	25700		423,577,089	146,197,947	Lleida	4,809,573	
F	77	585,407,882	yes	43001		411,191,029	12,584,219	Tarragona	818,283	
F	46	1,404,329,791	no	43001	1	411,191,029	12,584,219	Tarragona	818,283	
M	65	1,060,317,257	no	43003		411,191,029	12,584,219	Tarragona	818,283	
M	68	481,276,395	yes	43006	1	411,191,029	12,584,219	Tarragona	818,283	
M	68	1,117,298,912	no	43007		411,191,029	12,584,219	Tarragona	818,283	
F	24	1,437,236,355	no	43110		411,213,695	118,007,857	Tarragona	903,433	
M	71	1,218,780,767	no	43120		411,540,946	121,237,597	Tarragona	9,141,985	
M	75	1,043,649,061	yes	43120		411,540,946	121,237,597	Tarragona	9,141,985	
M	52	1,640,370,303	no	43130		411,191,029	12,584,219	Tarragona	818,283	
M	75	1,276,690,187	no	43130	1	411,191,029	12,584,219	Tarragona	818,283	
F	69	111,011,084	no	43140	1	411,872,881	121,069,822	Tarragona	89,955	
M	73	93,188,497	yes	43155		411,910,493	125,566,964	Tarragona	822,868	
F	71	1,384,780,308	no	43201		411,541,073	110,986,118	Tarragona	6,490,946	
M	65	1,154,740,532	no	43202	1	411,541,073	110,986,118	Tarragona	6,490,946	
M	47	1,150,120,469	yes	43202		411,541,073	110,986,118	Tarragona	6,490,946	
F	70	125,711,387	no	43204	1	411,541,073	110,986,118	Tarragona	6,490,946	
F	67	1,626,586,537	no	43205	1	411,424,109	11,041,302	Tarragona	756,836	
M	63	1,081,594,328	no	43430		414,008,278	105,182,973	Tarragona	7,028,451	
M	77	943,507,775	yes	43500		408,110,215	52,500,631	Tarragona	5,959,944	
M	57	1,277,107,434	no	43500		408,110,215	52,500,631	Tarragona	5,959,944	
F	64	139,135,785	no	43511		409,073,786	512,382	Tarragona	5,433,452	
M	55	1,174,812,412	no	43700	1	412,204,307	153,501,367	Tarragona	8,187,328	
M	56	1,171,813,693	no	43717		412,807,194	14,876,814	Tarragona	5,439,798	
M	75	725,858,079	yes	43820		412,017,176	156,834,858	Tarragona	7,796,463	
M	58	1,407	no	43820		412,017,176	156,834,858	Tarragona	7,796,463	
F	65	1,577	no	43820		412,017,176	156,834,858	Tarragona	7,796,463	
M	61	881,930,045	yes	43840		410,768,507	113,197,961	Tarragona	7,747,512	
M	76	1,073,654,851	yes	43860	1	408,850,022	80,032,416	Tarragona	5,596,739	
F	74	133,570,403	no	43880		412,204,307	153,501,367	Tarragona	8,187,328	
F	44	105,642,667	yes	43881		41,197,868	163,406,068	Tarragona	6,831,995	
F	64	1,288,684,987	no	43881		41,197,868	163,406,068	Tarragona	6,831,995	
M	46	486,414,985	yes	43895		408,125,297	70,877,628	Tarragona	6,106,034	
	64.8928571				67					
