# Supplementary material for: Harnessing PM2.5 Exposure Data to Predict Progression of Fibrotic Interstitial Lung Diseases Based on Telomere Length
Source: Front Med (Lausanne). 2022 May 12;9:871898. doi: 10.3389/fmed.2022.871898 (PMC9133476; doi:10.3389/fmed.2022.871898)
Supplement: Supplementary file 2 [file Data_Sheet_1.DOCX]

Shull Supplementary data 2
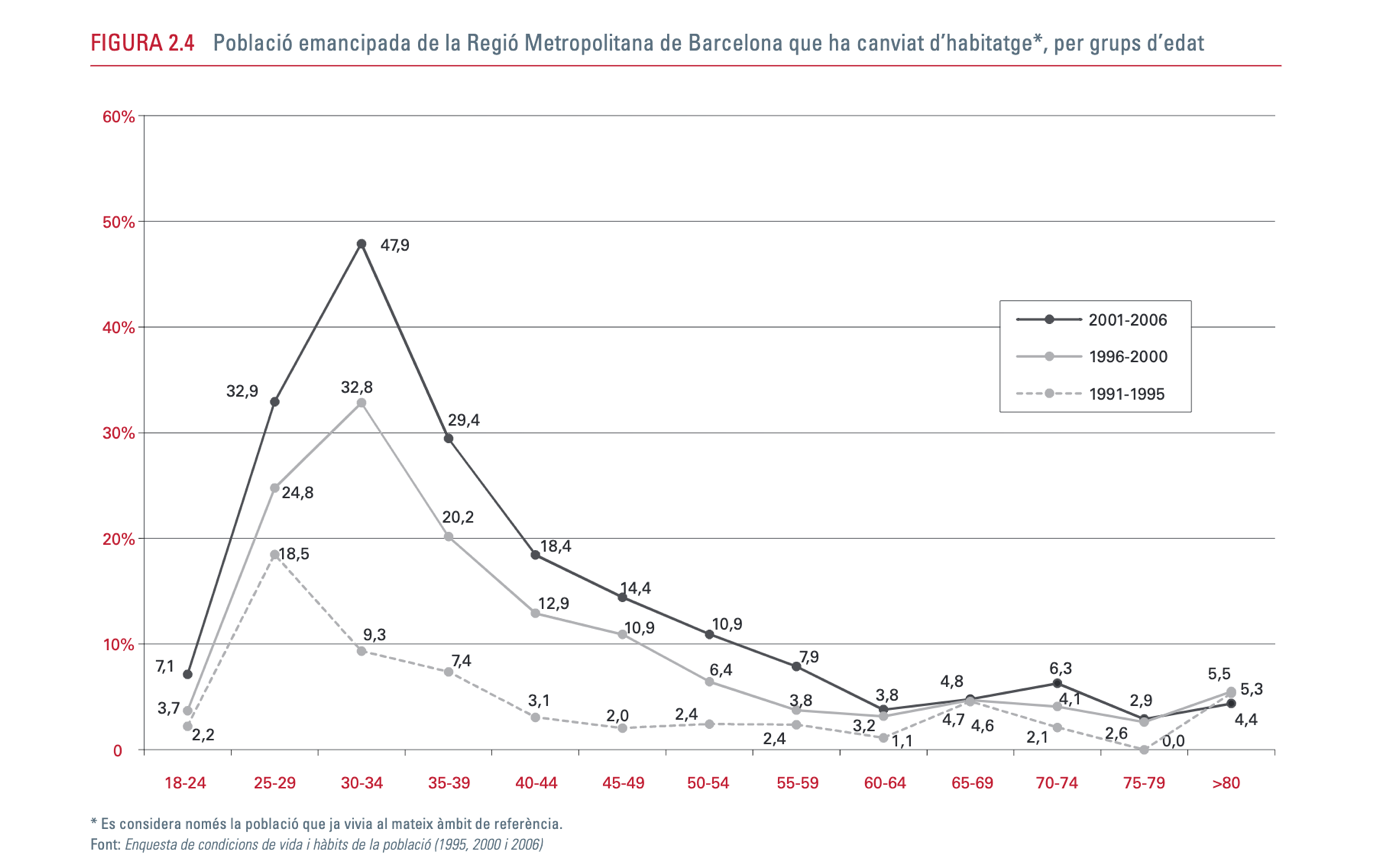


The Institute for Regional and Metropolitan Studies and the Barcelona City Council do frequent survey work of the population of Catalonia. The most recent results on housing and change of residence data were published in 2007 as the Survey of the living conditions and habits of the population of Catalonia(1).

The above graph labeled Figure 2.4 is from that publication. As it is in Catalan, the title of the graph is translated here: “Emancipated population of the Metropolitan Region of Barcelona that changed house, by age group(2).”

It can be seen that after the age of 50, the tendency to move is very low. This is due to a few factors, as pointed out in the survey publication, but the main reasons are stated by respondents as being “proximity and access to healthcare and shops” as well as “maintaining social relations in the area.”

1. Miralles-Guasch Carme, Donat Muñoz Carles BJ aum. Núm. 46 (2007): Habitatge i mobilitat residencial. Primers resultats de l’ECVHPC 2006 | Papers: Regió Metropolitana de Barcelona: Territori, estratègies, planejament. Habitat i mobilitat Resid Prim Result l’ECVHPC 2006 [Internet]. 2007 [cited 2022 Mar 13];115. Available from: https://raco.cat/index.php/PapersIERMB/issue/view/8487

2. Annex 1. Taules estadístiques complementàries. Pap Regió Metrop Barcelona Territ estratègies, planejament [Internet]. 2008 Jun 23;0(46 SE-Articles). Available from: https://raco.cat/index.php/PapersIERMB/article/view/104027

.
